# Detection of early Alzheimer's disease in MCI patients by the combination of MMSE and an episodic memory test

**DOI:** 10.1186/1471-2377-11-78

**Published:** 2011-06-24

**Authors:** Ana Pozueta, Eloy Rodríguez-Rodríguez, José Luis Vazquez-Higuera, Ignacio Mateo, Pascual Sánchez-Juan, Soraya González-Perez, José Berciano, Onofre Combarros

**Affiliations:** 1 Neurology Service, IFIMAV and CIBERNED, "Marqués de Valdecilla" University Hospital (University of Cantabria), Avda Valdecilla s/n, 39008-Santander, Spain; 2 AFAC (Alzheimer's Disease Association), Rosario de Acuña 7-bajo, 39008-Santander, Spain

## Abstract

**Background:**

Mild cognitive impairment (MCI) is a heterogeneous clinical entity that comprises the prodromal phase of Alzheimer's disease (Pr-AD). New biomarkers are useful in detecting Pr-AD, but they are not universally available. We aimed to investigate baseline clinical and neuropsychological variables that might predict progression from MCI to AD dementia.

**Methods:**

All patients underwent a complete clinical and neuropsychological evaluation at baseline and every 6 months during a two-year follow-up period, with 54 out of 109 MCI patients progressing to dementia (50 of them progressed to AD dementia), and 55 remaining as stable MCI (S-MCI).

**Results:**

A combination of MMSE and California Verbal Learning Test Long Delayed Total Recall (CVLT-LDTR) constituted the best predictive model: subjects scoring above 26/30 on MMSE and 4/16 on CVLT-LDTR had a negative predictive value of 93.93% at 2 years, whereas those subjects scoring below both of these cut-off scores had a positive predictive value of 80.95%.

**Conclusions:**

Pr-AD might be distinguished from S-MCI at baseline using the combination of MMSE and CVLT-LDTR. These two neuropsychological predictors are relatively brief and may be readily completed in non-specialist clinical settings.

## Background

Different biomarkers have been thoroughly studied in order to establish which ones best predict the progression from mild cognitive impairment (MCI) to Alzheimer's disease (AD) dementia. With current evidence, medial temporal lobe and hippocampal atrophy on MRI, glucose metabolism reduction measured by FDG-PET and CSF biomarkers reflecting AD pathology, are useful in detecting prodromal AD (Pr-AD) patients [1]. Nevertheless, these new biomarkers are still not universally available in routine clinical practice. A neuropsychological profile differentiating Pr-AD from stable MCI (S-MCI) has been investigated, and episodic memory impairment seems to be a strong predictor of progression to dementia [2-4], although it is not specific as some patients will not develop AD. Impairment in other cognitive domains (executive function and language) has been described in Pr-AD, but there are discrepancies about which additional impaired area will most improve sensitivity and specificity for detecting Pr-AD [5-7]. We aim to investigate the clinical variables and neuropsychological measures at the moment of MCI diagnosis, which will better predict the progression from MCI to early AD dementia in a two-year follow-up period.

## Methods

A total of 115 patients (63.3% females; mean age at diagnosis 74.4 years, SD 6.8; education, 63.3% primary school) were consecutively recruited from the Memory Clinic of University Hospital "Marqués de Valdecilla" (Santander, Spain) between April 2007 and April 2008. All of them initially fulfilled the Petersen criteria for MCI [8]. We excluded subjects who met criteria for dementia (DSM-IV), AD (NINCDS-ADRDA), depressive episode (IDC-10), subjects with significative cerebrovascular disease (Hachinski scale score ≥4), and those with any other medical or psychiatric identifiable cause accounting for their complaints. All patients underwent a complete clinical and neuropsychological evaluation at baseline and every 6 months during a two-year follow-up period. General cognitive function was assessed using MMSE, data on activities of daily living were collected using the Interview for Deterioration in Daily living activities in Dementia (IDDD), and symptoms of depression were measured using the Hamilton Rating Scale for Depression. Neuropsychological battery included test for the assessment of memory (California Verbal Learning Test-CVLT), language and semantic memory (15-items short-form of the Boston Naming Test, category fluency), praxis and visuospatial skills (Rey complex figure copy and WAIS block design subtest), attention and executive function (Symbol Digit Modalities Test, Trail Making part A and B, Stroop interference Test, Frontal Assessment Battery, category and letter fluency). A cognitive domain was judged as impaired when subjects scored 1.5 SD below values for age and education matched controls in at least one test. According to the results of the neuropsychological exploration, subjects were classified as: 1/ subjective memory complaints (SMC), patients performing normally on neuropsychological examination; 2/ pure amnestic MCI (a-MCI), patients fulfilling Petersen's criteria for amnestic MCI, with memory being the only affected domain; 3/ multidomain MCI (md-MCI), patients fulfilling a-MCI criteria and with one or more non-memory domain performance being under the cut-off value; 4/ non-amnesic MCI (na-MCI), patients with intact memory performance but scoring below the cut-off score on one or more non-memory tests.

The study was approved by the ethical committee of the University Hospital "Marqués de Valdecilla", and written informed consent was obtained from all the patients.

Statistical analyses were performed with SPSS software v.14.0. Differences in demographic, baseline clinical characteristics and neuropsychological measures between groups (S-MCI versus Pr-AD) were determined using Student's t-test for quantitative and χ^2 ^for categorical variables. We included in a logistic regression model all variables associated with disease progression (p < 0.05), plus gender, age and ApoE e4 status, in order to select those of them that were independent predictors of dementia. ROC curve analysis was performed to evaluate the discriminating power of the predictive model for the progression to dementia. The area under the curve (AUC) was used as a measure of the overall performance. Optimum cut-off points of selected variables were calculated by choosing the point on the ROC curve that maximized both sensitivity and specificity.

## Results

From the 115 patients included in the study, 6 patients were missed early in time and they did not complete the first year of follow-up, so they were excluded. The patients that ended the follow-up (n = 109) were classified in MCI subgroups according to their neuropsychological performance at baseline: 29 a-MCI (27%), 55 md-MCI (50%), 15 na-MCI (14%) and 10 SMC (9%). During the 2 years follow-up, 54 patients (49.54%) progressed to dementia, and 55 remained as S-MCI without progressing to dementia. Two patients were diagnosed as Lewy bodies dementia (LBD) and two as vascular dementia (VD), the remaining 50 patients being diagnosed as AD dementia. Given the initial classification in MCI subgroups, 62% (n = 18) of a-MCI and 58% (n = 32) of md-MCI patients evolved to AD dementia, whereas only 13% (n = 2) of na-MCI patients progressed to dementia (1 LBD and 1 VD). Among md-MCI subjects, one patient was diagnosed as VD and another was considered LBD, and none patient evolved to dementia in the SMC group.

We searched for differences in demographic, past medical history and neuropsychological assessments at baseline between S-MCI and Pr-AD (Table 1). Patients that evolved to non-AD dementia were excluded. In the univariate analysis, Pr-AD patients were significantly older than S-MCI, and Pr-AD scored significantly lower than S-MCI in MMSE, CVLT scores (short and long delay recall, both free and cued), category fluency and Frontal Battery Assessment (FAB). As expected, ApoE e4 allele was much more frequent in Pr-AD than in S-MCI. Conversely, active smoking and history of previous stroke were more frequent in S-MCI than Pr-AD, and S-MCI subjects scored slightly higher in Hamilton Rating Scale for depression. In our multivariate analysis, CVLT long delay total recall score (CVLT-LDTR) and MMSE turned out to be the only two variables independently associated to Pr-AD. In order to assess their discriminative power, we performed a ROC curve analysis that showed an area under the curve of 0.773 for MMSE (95% CI = 0.674-0.873, P < 0.001) and 0.775 for CVLT-LDTR (95% CI = 0.689-0.861, P < 0.001). The optimal thresholds for MMSE and CVLT-to predict progression from MCI to early AD were 26.5/30 and 4.5/16 respectively: those subjects that scored above 26/30 on MMSE and 4/16 on CVLT-LDTR had a negative predictive value of 93.93% at 2 years; inversely, those scoring below on both tests had a positive predictive value of 80.95%. For those subjects with just only one test below the cut-off score (Table 2), a firm prognosis could not be done as misclassifications were frequent. APOE ε4 status did not reach statistical significance (P = 0.168) when it was added to the multivariate predictive model.

**Table 1:** Comparison between prodromal AD and stable MCI according to baseline clinical and neuropsychological features

	Pr-AD n = 50	S-MCI n = 55	p-value		Pr-AD n = 50	S-MCI n = 55	p-value
Age	75.94 (6.05)	72.93 (7.30)	0.021	MMSE	25.92 (1.88)	27.78 (1.55)	**< 0.001**
Sex (% female)	59.37	64.58	0.575	WAT	16.87 (6.61)	17.32 (7.96)	0.778
Hamilton	3.92 (3.36)	6.32 (5.98)	0.033	Symbol-Digit	23.21 (10.86)	27.02 (10.86)	0.142
IDDD	41.09 (7.48)	38.11 (4.62)	0.071	CVLT-SDFR	1.52 (2.00)	4.55 (3.90)	< 0.001
Hachinski	1.97 (1.15)	2.27 (1.72)	0.373	CVLT-SDTR	3.78 (2.40)	6.45 (3.32)	< 0.001
Social extraction (% urban)	74.35	69.38	0.712	CVLT-LDFR	1.38 (1.86)	4.83 (4.03)	< 0.001
Marital status (% married)	56.41	71.42	0.143	CVLT-LDTR	3.29 (2.42)	6.58 (3.42)	**< 0.001**
Education (% primary school)	66.66	71.42	0.775	CVLT- Learning	12.83 (2.89)	13.77 (2.17)	0.054
HBP (%)	45.83	32.81	0.161	BNT	8.13 (2.58)	8.42 (2.46)	0.544
DM (%)	16.66	23.43	0.380	Rey figure copy	25.39 (10.71)	26.97 (9.00)	0.398
Smoking (% active)	6.25	25.00	0.009	WAIS block design	17.90 (8.24)	20.63 (7.89)	0.112
Alcohol (% active)	8.33	12.5	0.480	Stroop Test (interference)	-7.36 (8.32)	-5.71 (6.24)	0.233
Hypercholesterolemia (%)	33.33	31.25	0.815	TMT-A	111.05 (79.87)	88.38 (46.45)	0.083
Statins (%)	16.66	15.62	0.882	TMT-B	237.06 (120.56)	196.20 (98.85)	0.098
Ischemic cardiopathy (%)	4.16	10.93	0.192	Category fluency	10.88 (4.33)	12.87 (3.98)	0.017
Previous Stroke (%)	0	9.37	0.029	Phonemic fluency	5.89 (3.10)	6.98 (3.54)	0.099
Arterial ischemia (%)	0	1.56	0.384	FAB	12.94 (2.15)	14.44 (2.45)	0.004
Family History of Dementia (%)	31.25	28.12	0.720				
ApoE E4 carrier (%)	53.48	28.30	0.012				

Values are means (SD); p-value from univariate analysis, and in bold, those p-values which remained significant after the multivariate analysis adjusting for all associated variables plus age, sex and ApoeE e4 status; Pr-AD (prodromal AD): MCI patients that progressed to AD during the follow-up; S-MCI (stable MCI): MCI patients that did not progress to dementia; IDDD: Interview for Deterioration in Daily living activities in Dementia; HBP: High Blood Pressure; DM: Diabetes Mellitus; WAT: Word Accentuation Test; CVLT: California Verbal Learning Test; SDFR: Short Delayed Free Recall; SDTR: Short Delayed Total Recall (free and cued); LDFR: Long Delayed Free Recall; LDTR: Long Delayed Total Recall (free and cued); BNT: Boston Naming Test; TMT-A: Trail Making Test part A; TMT-B: Trail Making Test part B; FAB: Frontal Assessment Battery.

**Table 2:** Sensitivity, specifity, PPV and NPV of MMSE and CVLT-LDTR for predicting AD

n = 105	Sensitivity (%)	Specificity (%)	PPV (%)	NPV (%)
MMSE ≤ 26	64.10	79.59	71.42	73.58
CVLT-LDTR ≤ 4	72.91	70.31	64.81	77.58
MMSE ≤ 26 and/or CVLT-LDTR ≤ 4	94.80	63.26	67.27	93.93
MMSE ≤ 26 and CVLT-LDTR ≤ 4	43.58	91.83	80.95	67.16

PPV: positive predictive value; NPV: negative predictive value; CVLT-LDTR: California Verbal LearningTest Long Delayed Total Recall

## Discussion

As a whole, our progression rate is approximately 25% per year, within the range of conversion from MCI to AD previously reported [2,5,6,8-11]. In contrast with the literature indicating that md-MCI is the subgroup with the higher progression rate to dementia and that pure amnestic forms have lower rates of progression [6,10,12-14], in our series MCI of the amnestic type (regardless of other cognitive domains impairment) showed the higher risk of progression to dementia, with approximately 60% evolving to dementia during the 2 years follow-up. Amyloid imaging in MCI subtypes [15] showed that the highest proportion (approximately 80%) of amyloid-positive patients belonged to the md-MCI subgroup, although nearly 50% of a-MCI subjects were also amyloid-positive, thus reflecting subjacent AD pathology in a high proportion of a-MCI cases. Therefore, not only md-MCI but also a-MCI should be considered as high-risk of progression to dementia subgroups, and this is specially valid for those subjects with a poorer memory performance.

MMSE and CVLT-LDTR were the only measures that arose from multivariate analysis as independently associated with progression risk from MCI to early AD. According to our results, subjects scoring below 26/30 on MMSE and 4/16 on CVLT-LDTR constitute a MCI subgroup at high risk of progressing to early AD. MMSE is one of the most used tests for screening of cognitive impairment worldwide, and it has been reported that decline in MMSE starts approximately three years before the diagnosis of dementia [16]. Other tests used to determine general cognitive status (ADAS-cog, Addenbrooke's Cognitive Examination) have been reported as good predictors, independently [9] or associated with other neuropsychological measures [5,12]; however, these tests are not used so widespread in general neurology clinics. Episodic memory tests have been widely described as good predictors for AD in MCI subjects, although usually they lacked specificity [17]. Several types of memory test have been used [2-6,12,18], but comparisons between them are limited [17]. The long delay scores of verbal episodic memory tests that are based on learning across multiple trials (i.e. CVLT) seemed to provide the most sensitive index for initial diagnosis of MCI and for detecting subjects which most likely will evolve to AD, as their performance is highly dependent on entorhinal and hippocampal systems [17]. The amnestic syndrome of the hippocampal type described in AD [1] is specifically characterized by a decreased total recall due to little effect of cueing. This feature differs from that seen in frontotemporal dementia, vascular dementia, other causes of fronto-subcortical dementia and psychiatric diseases, such as depression, that usually benefit from cueing. Therefore, CVLT-LDTR is more sensitive to detect Pr-AD than other CVLT scores because it captures the characteristic AD amnestic syndrome. Semantic memory (measured usually with category fluency) and attention and executive functions (measured usually with TMT-A or Symbol Digit Modalities Test) have been described as predictors of clinical progression in several studies [5-7,13,19], mainly when combined with other episodic memory scores or general cognitive assessments. However, in our multivariate analysis, category fluency and FAB lost significance, indicating they did not add information to that given by MMSE and CVLT-LDTR.

## Conclusions

Pr-AD might be distinguished from S-MCI at baseline using the combination of MMSE and CVLT-LDTR. The clinical relevance of the findings is that these two neuropsychological predictors are relatively brief and may be readily completed in non-specialist clinical settings (where CSF biomarkers, MRI or PET are not available).

## Competing interests

The authors declare that they have no competing interests.

## Authors' contributions

AP performed the neuropsychological evaluations and reviewed critically the manuscript. ERR, JLVH and IM carried out the neurological evaluation and follow-up and reviewed critically the manuscript. PSJ performed the statistical analyses and reviewed critically the manuscript. SGP and JB reviewed critically the manuscript. OC drafted the manuscript and contributed to its final version. All authors read and approved the final manuscript.

## Pre-publication history

The pre-publication history for this paper can be accessed here:

http://www.biomedcentral.com/1471-2377/11/78/prepub
